# Functional and structural brain network development in children with attention deficit hyperactivity disorder

**DOI:** 10.1002/hbm.26288

**Published:** 2023-03-29

**Authors:** Shania Mereen Soman, Nandita Vijayakumar, Phoebe Thomson, Gareth Ball, Christian Hyde, Timothy J. Silk

**Affiliations:** ^1^ Centre for Social and Early Emotional Development and School of Psychology Deakin University Geelong Victoria 3125 Australia; ^2^ Child Mind Institute New York New York 10022 USA; ^3^ Department of Paediatrics University of Melbourne Parkville Victoria 3010 Australia; ^4^ Developmental Imaging Murdoch Children's Research Institute Flemington Road Parkville Victoria 3052 Australia

**Keywords:** attention deficit hyperactivity disorder, connectomes, diffusion weighted imaging, functional connectivity, graph theory, resting state functional magnetic resonance imaging, structural connectivity

## Abstract

Attention deficit hyperactivity disorder (ADHD) is a prevalent childhood neurodevelopmental disorder. Given the profound brain changes that occur during childhood and adolescence, it is important to examine longitudinal changes of both functional and structural brain connectivity across development in ADHD. This study aimed to examine the development of functional and structural connectivity in children with ADHD compared to controls using graph metrics. One hundred and seventy five individuals (91 children with ADHD and 84 non‐ADHD controls) participated in a longitudinal neuroimaging study with up to three waves. Graph metrics were derived from 370 resting state fMRI (197 Control, 173 ADHD) and 297 diffusion weighted imaging data (152 Control, 145 ADHD) acquired between the ages of 9 and 14. For functional connectivity, children with ADHD (compared to typically developing children) showed lower degree, local efficiency and betweenness centrality predominantly in parietal, temporal and visual cortices and higher degree, local efficiency and betweenness centrality in frontal, parietal, and temporal cortices. For structural connectivity, children with ADHD had lower local efficiency in parietal and temporal cortices and, higher degree and betweenness centrality in frontal, parietal and temporal cortices. Further, differential developmental trajectories of functional and structural connectivity for graph measures were observed in higher‐order cognitive and sensory regions. Our findings show that topology of functional and structural connectomes matures differently between typically developing controls and children with ADHD during childhood and adolescence. Specifically, functional and structural neural circuits associated with sensory and various higher order cognitive functions are altered in children with ADHD.

## Graphical abstract

This study explores longitudinal development of both functional and structural connectivity across late childhood and in children with ADHD and typically developing controls. We demonstrated that topology of functional and structural connectomes matures differently between ADHD and controls. Specifically, functional and structural neural circuits associated with sensory and various higher order cognitive functions are altered in children with ADHD.

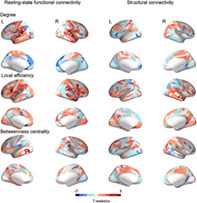

## 1 INTRODUCTION

Attention deficit hyperactivity disorder (ADHD) is one of the most common neurodevelopmental disorders, characterized by symptoms of age‐inappropriate attention and/or hyperactivity/impulsivity (American Psychiatric Association, 2013; Faraone et al., 2003). A growing literature examining the brain systems underlying ADHD have identified differences in the structural and functional connectivity of spatially distributed, but interconnected neural networks, as well as various behaviours associated with these networks (Bos et al., 2017; Griffiths et al., 2021; Sörös et al., 2019). However, much of this research has been conducted in cross‐sectional samples, and as such, much less is known about longitudinal maturation of brain connectivity patterns in ADHD during childhood and adolescence.

Our brain is a complex integrated network that coordinates billions of neurons (Bullmore & Sporns, 2009). Macroscopically, the large number of interconnected neurons is organized into different brain structures that perform various functions together (Bullmore & Sporns, 2009). Two modalities of magnetic resonance imaging (MRI), resting state functional magnetic resonance imaging (rs‐fMRI) and diffusion MRI (dMRI), have been widely used to inform our understanding of functional and structural connectivity of the brain at a macroscopic scale, as well as altered patterns implicated in various neurodevelopmental disorders (Bos et al., 2017; d'Albis et al., 2018; Lau et al., 2019; Sutcubasi et al., 2020). Rs‐fMRI estimates neural activity indirectly by measuring spontaneous fluctuations of oxygenated blood in the brain at rest (Logothetis, 2002), while measures derived from dMRI can be used to estimate white matter microstructure and the anatomical connectivity of different brain regions (Bergamino et al., 2020). Many cross‐sectional rs‐fMRI studies in children with ADHD have reported impaired connectivity of various resting state networks that support higher order cognition and affective systems (i.e., default mode network (DMN) (Cortese et al., 2012, 2021; Fair et al., 2010; Posner et al., 2014; Qiu et al., 2011), ventral attention network (VAN) (Cortese et al., 2012), executive control networks (Cao et al., 2009; Cortese et al., 2012; Fair et al., 2010; Sun et al., 2012), somatomotor network (Cortese et al., 2012) and limbic network (Cao et al., 2009; Castellanos et al., 2009; Posner et al., 2013; Tian et al., 2006)). In parallel, cross‐sectional dMRI studies have identified differences in white matter tracts linking frontal, parietal and cerebellar regions (Ameis et al., 2016; Aoki et al., 2018; Cao et al., 2010; Castellanos & Proal, 2012; Chiang et al., 2015, 2016; Connaughton et al., 2022; Durston et al., 2011; Fuelscher et al., 2021; Gau et al., 2015; Hamilton et al., 2008; King et al., 2015; Lin et al., 2014; Nagel et al., 2011; Pastura et al., 2016; Pavuluri et al., 2009; Qiu et al., 2011; Shang et al., 2013; Silk et al., 2009; Tung et al., 2021; Wu et al., 2017; Wu et al., 2020), with microstructural properties of these tracts linked to poor visual and sustained attention in children with ADHD (Griffiths et al., 2021; Witt & Stevens, 2015). Collectively, findings suggest that ADHD is characterized by anomalies in distributed networks of both functional and structural connectivity. However, since most of these studies are cross‐sectional it is not clear whether the reported differences reflect different trajectories of structural and functional network development between groups over time.

A recent longitudinal study found that resting‐state functional connectivity within the cingulo‐opercular network over development was associated with psychostimulant treatment in ADHD (Norman et al., 2021). Additionally, few recent longitudinal studies in dMRI investigated longitudinal changes of ADHD symptoms with white matter microstructure (Damatac et al., 2022; Thomson et al., 2022). Damatac and colleagues revealed that improvement in hyperactivity‐impulsivity symptoms over time was linked with more fibre density in left cortico‐spinal tract (CST) and improvement in combined symptoms was associated with greater fibre cross‐section in left CST (Damatac et al., 2022). Thomson and colleagues reported that greater fibre cross‐section and fibre density in SLF is associated with better sustained attention across ADHD and controls (Thomson et al., 2022). These studies indicate that functional and structural networks undergo changes across development in children with ADHD. However, as these longitudinal studies has focused on how specific symptoms relates to certain resting‐state functional networks and tracts of interest, further research is needed to understand the potential role of other structural and functional networks over development in children with ADHD.

Graph theory permits the representation of the brain as a complex network, or “connectome” (Sporns, 2012), to facilitate a better understanding of brain function and organization. Graph theory can be applied to brain networks to identify when developmental differences in in network connectivity may lead to variability in neurodevelopmental outcomes (Fornito et al., 2015; Sporns et al., 2005; Vecchio et al., 2017). When applied to the brain's connectome, graph theory provides insight into the architecture of functional and structural brain networks (Sporns, 2018). This technique has been widely used in functional (Buckner et al., 2009; Stam et al., 2007; Supekar et al., 2009) and structural connectivity (Beare et al., 2017; Fortanier et al., 2019; Giacopelli et al., 2020; Supekar et al., 2009) studies where the nodes of the graph represent different grey matter regions of the brain and are connected by edges that represent the correlation between brain regions or the anatomical connections (properties of white matter tracts) between brain regions for functional and structural brain networks, respectively (Kerepesi et al., 2016).

Families of graph theory metrics offer different perspectives on network connectivity. Efficiency measures, such as the global and local efficiency, refer to how efficiently information can be transferred between nodes across a network; centrality measures, such as degree and betweenness centrality, reflect the relative importance of a given node to the transmission of information across a network (Achard & Bullmore, 2007; Bullmore & Sporns, 2009; Sporns, 2018). Global and local efficiency measures have been widely used in cross‐sectional studies to describe age‐related differences in the integration and segregation of brain networks (Cao, Wang, et al., 2014; Dennis et al., 2013; Geerligs et al., 2015; Gozdas et al., 2019; Justina et al., 2015; Wu et al., 2013). A high global efficiency (GE) indicates the capacity for rapid exchange of information across distributed brain regions (Bullmore & Sporns, 2009), while local efficiency refers to the efficiency of each region (node) and measures information transfer in the immediate neighbourhood of a given region (Oldham & Fornito, 2018). Other widely used basic graph measures include degree centrality, which identifies important network nodes based on the number of its connections (Oldham & Fornito, 2018); and betweenness centrality, that measures how important a brain region or node's connections are in the transmission of information across the brain network (Oldham & Fornito, 2018).

Graph metrics of structural and functional connectivity in children with ADHD predominantly encompass differences in degree, local efficiency, and betweenness centrality relative to typically developing children (Chen et al., 2019; dos Santos et al., 2014; Griffiths et al., 2021; Justina et al., 2015; Silk et al., 2019). Resting‐state functional connectivity studies in children with ADHD have reported alterations in local efficiency in mixed directions, but consistently implicate regions within the frontal cortex that subserve executive functions (Chen et al., 2019; Wang et al., 2009). For degree, children with ADHD are reported to exhibit lower connectivity in regions of the dorsal attention network (DAN) and default mode network (DMN), and higher connectivity in the limbic network (Di Martino et al., 2013; Tomasi & Volkow, 2012). While some have not identified group differences in betweenness centrality (Chen et al., 2019), others have shown it has strong predictive accuracy (73%) for the classification of ADHD (dos Santos et al., 2014). Compared to functional connectivity, very few studies have used graph measures to examine the brain's structural topology in children with ADHD (Beare et al., 2017; Cao et al., 2013; Griffiths et al., 2021; Qian et al., 2021). These have observed higher local efficiency in frontal and parietal regions in ADHD compared to typically developing children (Beare et al., 2017; Cao et al., 2013) and lower degree in limbic network in ADHD (Qian et al., 2021). Taken together, prior findings indicate alterations in network architecture of functional and structural connectivity in children with ADHD relative to typically developing children, primarily implicating regions involved in attention, higher‐order cognition, and affective processes. However, there has not been any research into the potentially different developmental trajectories of network architecture of functional and structural connectivity in children with ADHD. Moreover, several cross‐sectional studies have identified linear, nonlinear and stable changes in structural and functional degree, local efficiency and betweenness centrality during typical development (Chen et al., 2013; Dennis & Thompson, 2022). Decreases with age in the frontal cortex and increases with age in the temporal cortex have been observed for degree, local efficiency and betweenness centrality (Cao, Wang, et al., 2014; Dennis & Thompson, 2022). This could indicate the refinement of networks due to the pruning of fibres and may point to regional variation in temporal processes during typical development in various brain regions (Dennis & Thompson, 2022; Gogtay & Thompson, 2010). Overall, findings suggest that typical brain development is characterized by changes in the network topology of the brain, and that children with ADHD may exhibit atypical structural and functional topology. However, due to the lack of longitudinal studies, it is unclear whether these differences in structural and functional topology reflect atypical neurodevelopment in children with ADHD.

The objective of the current study was to investigate longitudinal changes in functional and structural connectivity in children with ADHD and typically developing counterparts. We used graph measures of local efficiency, degree and betweenness centrality, which have predominantly been reported in prior cross‐sectional connectivity studies on ADHD. We hypothesized group differences in degree, local efficiency and between centrality in regions that support attention, higher‐order cognitive and affective processes. We also hypothesized that children with ADHD will show differential developmental trajectory in regions of frontal, temporal, and visual regions of the brain for local efficiency, degree and betweenness centrality. However, we did not have expectations regarding directionality of these differences given mixed findings in prior cross‐sectional research on group differences, and the novelty of the longitudinal analyses. The novelty of this study is the examination of the development of both functional and structural networks using the same atlas (defined from structure and function), and using the same graph theory metrics.

## 2 METHOD

### 2.1 Participants

The current study comprised a community sample of 175 children with and without ADHD (91 children with ADHD and 84 non‐ADHD controls) between the ages of 9 and 14 years. Participants were recruited into the longitudinal neuroimaging project, Neuroimaging of the Children's Attention Project (NICAP) (Silk et al., 2016), in Melbourne, Australia. Each participant underwent up to three waves of repeated MRI scans with approximately 18‐month intervals. All children were screened using parent and teacher reports on Conners 3 ADHD Index and diagnostically confirmed using a parent face‐to‐face diagnostic interview (NIMH Diagnostic Interview Schedule for Children IV [DISC‐IV]). All the details regarding participants recruitment and screening can be found in (Sciberras et al., 2013). The diagnostic confirmation was initially done at recruitment (3 years before neuroimaging baseline) and was then repeated at the baseline wave of neuroimaging assessments. Children with a childhood history of ADHD (i.e., met criteria for ADHD at either recruitment or baseline) were included in the ADHD group. The control group similarly had to screen negative to parent and teacher Conners 3 ADHD Index, and not meet criteria for ADHD in the diagnostic interview at any waves. Written informed consent, in line with approval from the Human Research Ethics Committee of the Royal Children's Hospital, was obtained from all parents (Soman et al., 2022).

After quality control of imaging data (detailed below), functional scans missing a field map (*N* = 25 [ADHD = 12]), those with excessive head motion (greater than 0.5 mm of framewise displacement (FD) (Power et al., 2012), *N* = 10 (ADHD = 6)) and structural scans with low‐quality DWI data (*N* = 45 [ADHD = 18]) were excluded. There was no difference between the included and excluded in terms of the age distribution of control or ADHD participants. However, those children with ADHD who were excluded had more severe ADHD symptoms than included ADHD subjects (*p* < 0.05).

**FIGURE 1:**
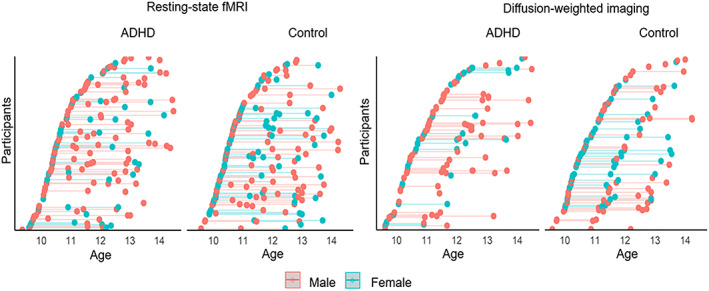
Distribution of ADHD and control participants who met inclusion criteria for resting‐state fMRI and DWI analyses.

The final sample comprised 372 functional scans (195 Control, 177 ADHD) and 297 structural scans (152 Control, 145 ADHD) across the three assessment waves (see Tables 1 and 2 and Figure 1 for further details). At any given wave 10%–20% of the ADHD group were taking medication related to their diagnoses, and of this subset, medications comprised methylphenidate: 76%–86%, atomoxetine: 0%–10%, lisdexamfetamine: 10%–17%. In addition to one of the former, 23%–29% were concurrently taking clonidine or fluoxetine.

**TABLE 1:** Demographic characteristics of participants in functional connectivity analyses.

	ADHD	Control	Difference
Participants wave 1 (% male)	68 (73%)	72 (49%)	*χ* ^2^ = 0.11
Participants wave 2 (% male)	66 (72%)	70 (56%)	*χ* ^2^ = 1.44
Participants wave 3 (% male)	43 (67%)	53 (62%)	*χ* ^2^ = 0.16
Age wave 1, Mean (SD)	10.40 (0.57)	10.45 (0.47)	*t* = 0.98
Age wave 2, Mean (SD)	11.63 (0.61)	11.72 (0.64)	*t* = −0.14
Age wave 3, Mean (SD)	13.24 (0.63)	13.09 (0.66)	*t* = −0.73
Mean head motion wave 1, Mean (SD)	0.19 (0.16)	0.18 (0.38)	*t* = 0.11
Mean head motion wave 2, Mean (SD)	0.16 (0.11)	0.13 (0.09)	*t* = −1.51
Mean head motion wave 3, Mean (SD)	0.11 (0.06)	0.10 (0.05)	*t* = −1.06
DSM inattentive symptoms, Mean (SD)	6.77 (1.74)	0.85 (1.33)	*t* = −22.84*
DSM hyperactive/impulsive symptoms, Mean (SD)	5.74 (2.59)	1.18 (1.48)	*t* = −12.54*
Conners 3 ADHD index, Mean (SD)	13.51 (4.52)	1.15 (1.93)	*t* = −20.95*
ADHD medication wave 1 (%)	11 (16%)	‐	‐
ADHD medication wave 2 (%)	12 (18%)	‐	‐
ADHD medication wave 3 (%)	6 (13%)	‐	‐

* *p* < 0.0001.

Abbreviation: SD, standard deviation.

**TABLE 2:** Demographic characteristics of participants in structural connectivity analyses.

	ADHD	Control	Difference
Participants wave 1 (% male)	54 (63%)	60 (45%)	*χ* ^2^ = 0.31
Participants wave 2 (% male)	53 (75%)	52 (56%)	*χ* ^2^ = 0.00
Participants wave 3 (% male)	38 (45%)	40 (58%)	*χ* ^2^ = 0.05
Age wave 1, Mean (SD)	10.36 (0.46)	10.37 (0.46)	*t* = 0.02
Age wave 2, Mean (SD)	11.77 (0.55)	11.70 (0.53)	*t* = −2.11
Age wave 3, Mean (SD)	13.24 (0.63)	13.22 (0.62)	*t* = −0.95
Mean head motion wave 1, Mean (SD)	0.96 (0.16)	0.92 (0.10)	*t* = −1.73
Mean head motion wave 2, Mean (SD)	0.77 (0.17)	0.81 (0.17)	*t* = 1.83
Mean head motion wave 3, Mean (SD)	0.31 (0.09)	0.29 (0.05)	*t* = −1.28
DSM inattentive symptoms, Mean (SD)	7.00 (1.61)	0.87 (1.52)	*t* = 21.00*
DSM hyperactive/impulsive symptoms, Mean (SD)	5.79 (2.20)	1.08 (1.74)	*t* = 11.91*
Conner 3 ADHD index, Mean (SD)	14.00 (4.12)	1.21 (2.36)	*t* = 19.11*
ADHD medication wave 1 (%)	11 (20%)	‐	‐
ADHD medication wave 2 (%)	11 (20%)	‐	‐
ADHD medication wave 3 (%)	4 (10%)	‐	‐

* *p* < 0.0001.

Abbreviation: SD, standard deviation.

### 2.2 MRI acquisition

All participants underwent a 30 min mock (practice) scanner session to get familiarized to the MRI environment. Subsequently, MRI scans were acquired using a 3‐Tesla Siemens scanner at a single site. However, waves 1 and 2 were collected on a TIM Trio scanner and wave 3 was collected after an upgrade to a MAGNETOM Prisma scanner (note that scanner upgrade was accounted for within statistical modelling). Using a 32‐channel head coil, functional images were acquired using multi‐band accelerated EPI sequences (MB3), with the following parameters: repetition time (TR) = 1500 ms, echo time (TE) = 33 ms, field of view (FOV) = 255 × 255 mm, flip angle (FA) = 85°, 60 axial slices, matrix size = 104 × 104, voxel size = 2.5 mm^3^, and 250 volumes acquired covering the whole brain in a 6 min 33 s sequence. Participants were instructed to keep their eyes open and to look at a fixation cross. High Angular Resolution Diffusion Imaging (HARDI) data were acquired using a multi‐band factor of 3 with the following parameters: *b* = 2800 s/mm^2^, 63 slices, matrix size = 110 × 100, voxel size = 2.4 mm^3^, FOV = 260 × 260 mm, bandwidth = 1748 Hz, acquisition time = 3 min 57 s. T1 weighted images were acquired using a multi‐echo magnetization prepared rapid gradient‐echo (MEMPRAGE) sequence along with navigator based prospective motion correction with the parameters: TR = 2530 ms, TE = 1.77, 3.51, 5.32 and 7.2 ms, FOV = 230 × 230 mm, FA = 7°, axial slices = 176, matrix size = 256 × 232, voxel size = 0.9 mm^3^, acquisition time = 6 min 52 s (Soman et al., 2022).

### 2.3 Preprocessing of functional data

Resting state fMRI images were preprocessed using FSL 5.0.9 (http://fsl.fmrib.ox.ac.uk/fsl/fslwiki). Standard preprocessing steps such as discarding of four initial volumes to account for initial signal inhomogeneity, motion correction using MCFLIRT (FMRIB's Linear Registration Tool), B0 unwarping, spatial smoothing using 5 mm FWHM, spatial normalization to the MNI template using a 12‐parameter affine transformation and registration of fMRI images to MNI space via high resolution T1 images using FSL FLIRT and FNIRT were undertaken (Anderson et al., 2007; Jenkinson et al., 2002; Soman et al., 2022). Further, each preprocessed image was decomposed using Multivariate Exploratory Linear Decomposition into Independent Components (MELODIC) in FSL. Following MELODIC, the resulting components from 20 subjects were manually classified as signal or noise based on previously mentioned criteria (Griffanti et al., 2014, 2017). FIX (FMRIB's ICA‐based Xnoisefier) (Salimi‐Khorshidi et al., 2014) classifier was trained using these classifications. FIX was then run on all single‐session MELODIC output to auto‐classify Independent Component Analysis (ICA) components into good versus bad components and denoise the data (Soman et al., 2022).

### 2.4 Preprocessing of structural data

All the steps for preprocessing diffusion data were undertaken with MRtrix3Tissue, a fork of the MRtrix software (Tournier et al., 2019). Raw diffusion images were pre‐processed using commands in MRtrix which are interfaced with external software programs such as FSL (Smith et al., 2004) and ANTS (Avants et al., 2009). All the participants underwent preprocessing steps of denoising (Veraart et al., 2016), Gibbs unringing (Kellner et al., 2016), correction for eddy current, motion‐distortion (Tustison et al., 2010) and bias field (Tustison et al., 2010), and brain mask estimation. After pre‐processing the structural data, response functions (Dhollander et al., 2019) for white matter, grey matter, cereberospinal fluid and the orientation of the fibres in each voxel were estimated (Fibre Orientation Distribution[FOD]) (Tournier et al., 2007). Further, global intensity differences among the data were corrected using intensity normalization.

### 2.5 Functional and structural connectome

For each subject, at each wave, functional and structural cortical connectivity matrices were defined using the multi‐modal parcellation of human cerebral cortex (HCP‐MMP) atlas (360 distinct regions) (Glasser et al., 2016). The volumetric version of the HCP‐MMP atlas available in AFNI (Cox, 1996) was used for the analysis, and the atlas was converted and mapped into each subject's surface space using Freesurfer (Desikan et al., 2006; Fischl et al., 1999). For the functional cortical connectivity (FC) matrix, Pearson correlation coefficient between each pair of ROIs was calculated using the Connectivity toolbox (CONN20b), resulting in a connectivity matrix of size 360 × 360. Structural cortical connectivity (SC) matrix for each subject in each wave was created by following the steps for estimating whole brain tractogram outlined in Basic and Advanced Tractography (BATMAN) (Tahedl, 2020). Streamlines were created using anatomically constrained tractography (Robert et al., 2012), and spherical‐deconvolution informed filtering of tracks (SIFT) (Smith et al., 2013). Further, the SC for each subject at each wave was created by scaling contribution of each streamline to the connectome edge by the inverse of the two node volumes (Glasser et al., 2016), with a symmetric format and diagonals set to zero. The functional and structural cortical connectivity matrices were converted into binary and undirected matrices.

A thresholding procedure was applied to functional and structural cortical connectivity matrices to eliminate the confounding effects of spurious relationships in interregional connectivity before performing topological characterization. For functional cortical connectivity, a threshold was selected based on the cost of functional brain network (Fornito et al., 2010; Stam & Reijneveld, 2007). A threshold value of 0.15 was chosen from a range of thresholds (0–0.5) as this resulted in networks with “small‐world” properties (i.e., comparatively high global efficiency compared to lattices, and comparatively high local efficiency compared to random graphs) (refer Figure S3 for details). For reproducibility we also conducted additional longitudinal analysis for graph measures of degree, local efficiency and betweenness centrality at thresholds below (0.1) and above (0.2) our primary threshold. The significant results observed for group differences and differential developmental trajectories for all the three thresholds (0.1, 0.15, 0.2) are present across similar areas of the cortex (Tables S4–S7). Structural cortical connectivity matrices were thresholded using consistency‐based thresholding at the 75th percentile for edge weight coefficient of variation to reduce the influence of false positives and false negatives, and nodes with zero connections after thresholding were excluded, as suggested in prior research (Graham et al., 2020; Roberts et al., 2017).

### 2.6 Graph analysis of connectomes

Graph theoretical measures for functional and structural cortical connectivity were extracted using the Brain Connectivity Toolbox (Rubinov & Sporns, 2010). Three graph measures describing regional cortical properties were examined. For a given node, *i*: Local efficiency (LE) is the inverse of the average shortest path connecting all neighbouring nodes of node *i*, excluding node *i*. The LE of node *i* in a network G is measured as $E_{\mathrm{loc}}=\frac{1}{N}-1\sum j\neq i\in G\frac{1}{d_{\mathrm{ij}}}$, where $d_{\mathrm{ij}}$ is the shortest path length between node *i* and node *j*. Degree sums the number of edges connected to node *i*. Betweenness Centrality (BC) is calculated based on the shortest paths between pairs of nodes in the network that pass‐through node *i*. BC is calculated as $B_{i}=\sum a\neq i\neq b\in G\frac{\sigma_{\mathrm{ab}}\left(i\right)}{\sigma_{\mathrm{ab}}}$, where $\sigma_{\mathrm{ab}}$ is the number of shortest paths from node *a* to node *b* and $\sigma_{\mathrm{ab}}\left(i\right)$ is the number of shortest paths from node *a* to *b* that pass through node *i*. We also measured global efficiency (GE), which examines the efficiency of information transfer averaged across all nodes. For a network G, global efficiency is calculated as $E_{\text{glob}}=\frac{1}{N}\left(N-1\right)\sum i\neq j\in G\frac{1}{d_{\mathrm{ij}}}$, where $d_{\mathrm{ij}}$ is the shortest path length between node *i* and node *j*.

### 2.7 Longitudinal modelling of graph theoretical measures

The developmental changes of each graph theoretical measure in children with ADHD versus typically developing controls were examined with Generalized Additive Mixed Models (GAMM), using the “mgcv” package (Wood, 2017) in R (Team RDC, 2010), which are well‐suited to identify nonlinear longitudinal trends without the need to specify a functional form (linear, quadratic, etc.). The following four models were examined separately for each graph measure of functional and structural connectivity networks: (i) a null model without predictors, (ii) main smooth effect of age (refer to supplementary materials for results [Figures S1 and S2 and Table S1]), (iii) main effect of group (ADHD vs. Controls), and (iv) a smooth model examining the interaction of group and age. All models included frame‐wise displacement of functional or structural data, scanner (pre vs. post upgrade), medication status, and sex as covariates. To predict each graph measure, the basis dimension for the smooth term was set to 4 (maximum degrees of freedom for smooth term) as recommended by Wood (van Duijenvervoode et al., 2019). Each model was fit using maximum likelihood function and models with nested terms compared to identify the best‐fitting model. Akaike Information Criterion (AIC) was used to identify the best fitting model, where significant models (*p <* 0.05) with more than 2 AIC units less than other nested models were selected as the best‐fitting model (Bozdogan, 1987). Further, a False Discovery Rate (FDR) of 0.05 was used to determine the statistical significance of coefficients across 360 regions. Whole brain maps and trajectory plots were created using Pysurfer v0.10.10 (https://pysurfer.github.io/) and Rstudio (Team RDC, 2010), respectively.

## 3 RESULTS

No significant effects were identified across any statistical analyses for global efficiency.

### 3.1 Group differences: Main effect of group

#### 3.1.1 Functional cortical connectivity

**FIGURE 2:**
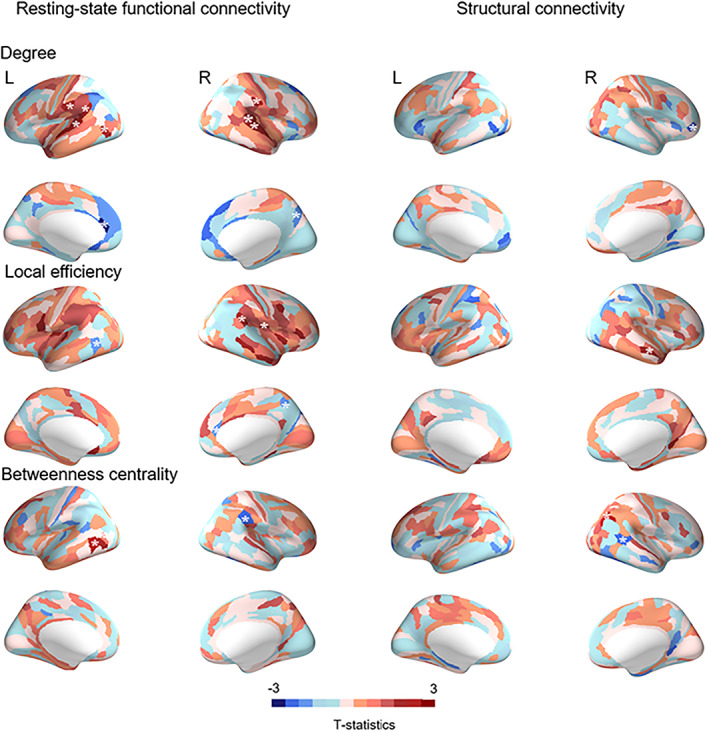
Group differences between ADHD and controls in functional and structural connectivity. Blue indicates stronger connectivity in ADHD and red indicates stronger connectivity in controls. “*” Indicates regions that survived FDR correction (*p* < 0.05).

Children with ADHD showed lower degree in bilateral inferior parietal cortex, superior temporal gyrus and left visual cortex, but higher degree in the left anterior cingulate, relative to typically developing children (Figure 2 and Table S2). Children with ADHD also showed lower LE in the right inferior parietal and insular cortex, but higher LE in the left inferior temporal, right precuneus and ventral anterior cingulate cortices (Figure 2 and Table S2). Furthermore, children with ADHD showed lower BC in left inferior temporal cortex and higher BC in the right inferior parietal cortex compared to typically developing children (Figure 2 and Table S2).

#### 3.1.2 Structural cortical connectivity

Children with ADHD had higher degree in the right dorsal posterior cingulate area (BA 31) and right frontal opercular cortex compared to typically developing children (Figure 2 and Table S2), as well as significantly lower LE in right dorsal posterior cingulate area (BA 31) and right middle temporal cortex (Figure 2 and Table S2). Children with ADHD also showed lower BC in right angular gyrus (BA 39/inferior parietal cortex) and higher BC in right middle temporal cortex relative to typically developing children (Figure 2 and Table S2).

### 3.2 Differential developmental trajectories: Group‐by‐age interaction

#### 3.2.1 Functional cortical connectivity

**FIGURE 3:**
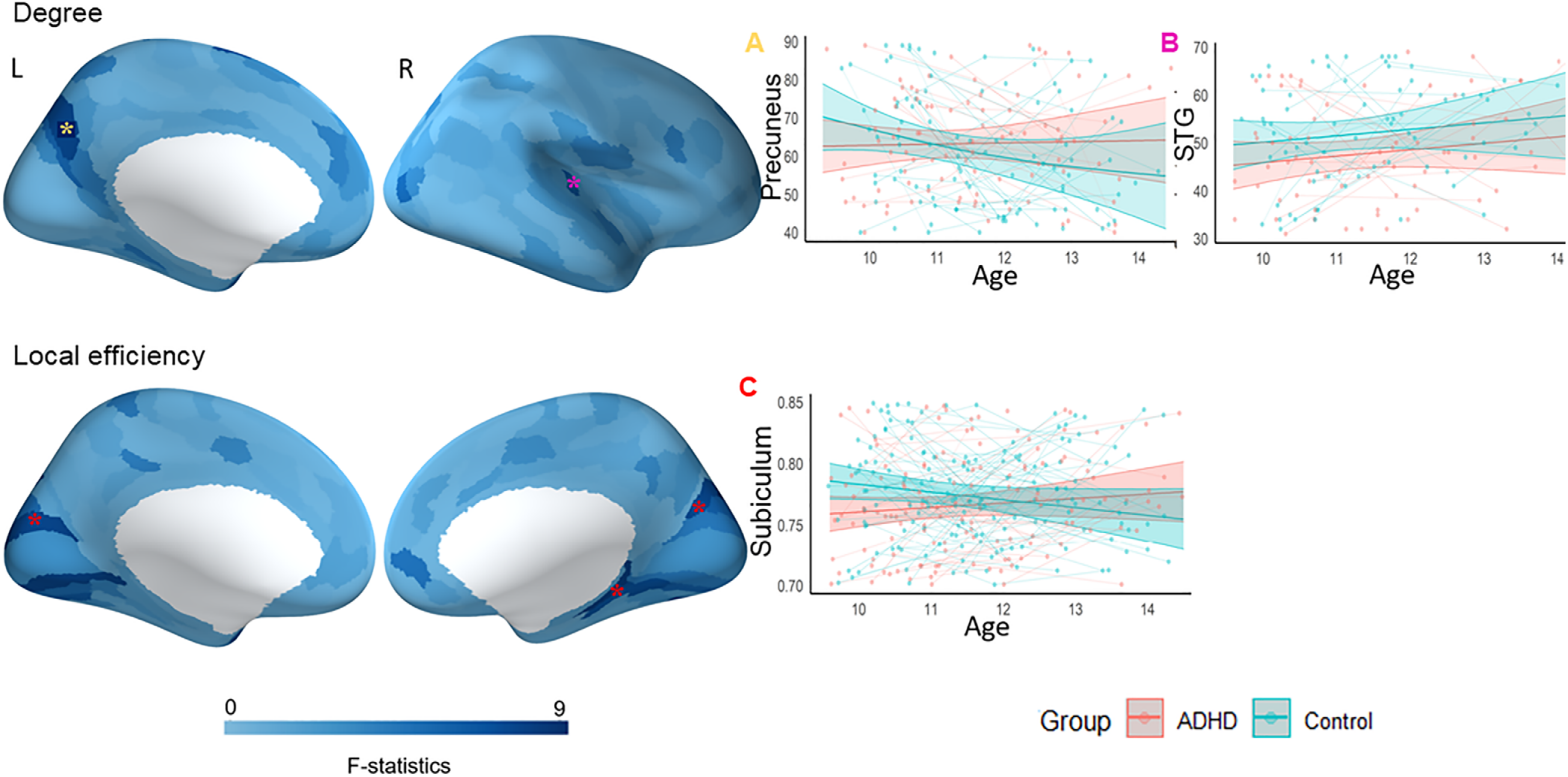
Group differences in developmental trajectories of functional connectivity (i.e., group x age interaction). “*” Indicates regions that survived FDR correction (*p* < 0.05). STG‐superior temporal gyrus. Plot A depicts the developmental trajectory of brain region marked with yellow asterisk (left precuneus), and plot B depicts the developmental trajectory of the brain regions marked with magenta asterisk (right STG). Regions marked with red asterisk showed similar pattern of developmental trajectory (bilateral visual cortex and right subiculum) and plot C depicts the developmental trajectory of a brain region (right subiculum) marked with red asterisk.

Children with ADHD showed greater increases in degree, compared to typically developing children, in the right superior temporal gyrus from late childhood to early adolescence. (Figure 3 and Table S3). Further, children with ADHD exhibited a stable trajectory for degree of the left precuneus, compared to reductions observed in typically developing children. For LE, children with ADHD showed a stable trajectory in the left visual association cortex (BA 18), right visual cortex, and right subiculum (inferior part of hippocampus) whereas typically developing children exhibited reductions (Figure 3 and Table S3). No significant interaction between age and group was observed for BC.

#### 3.2.2 Structural cortical connectivity

**FIGURE 4:**
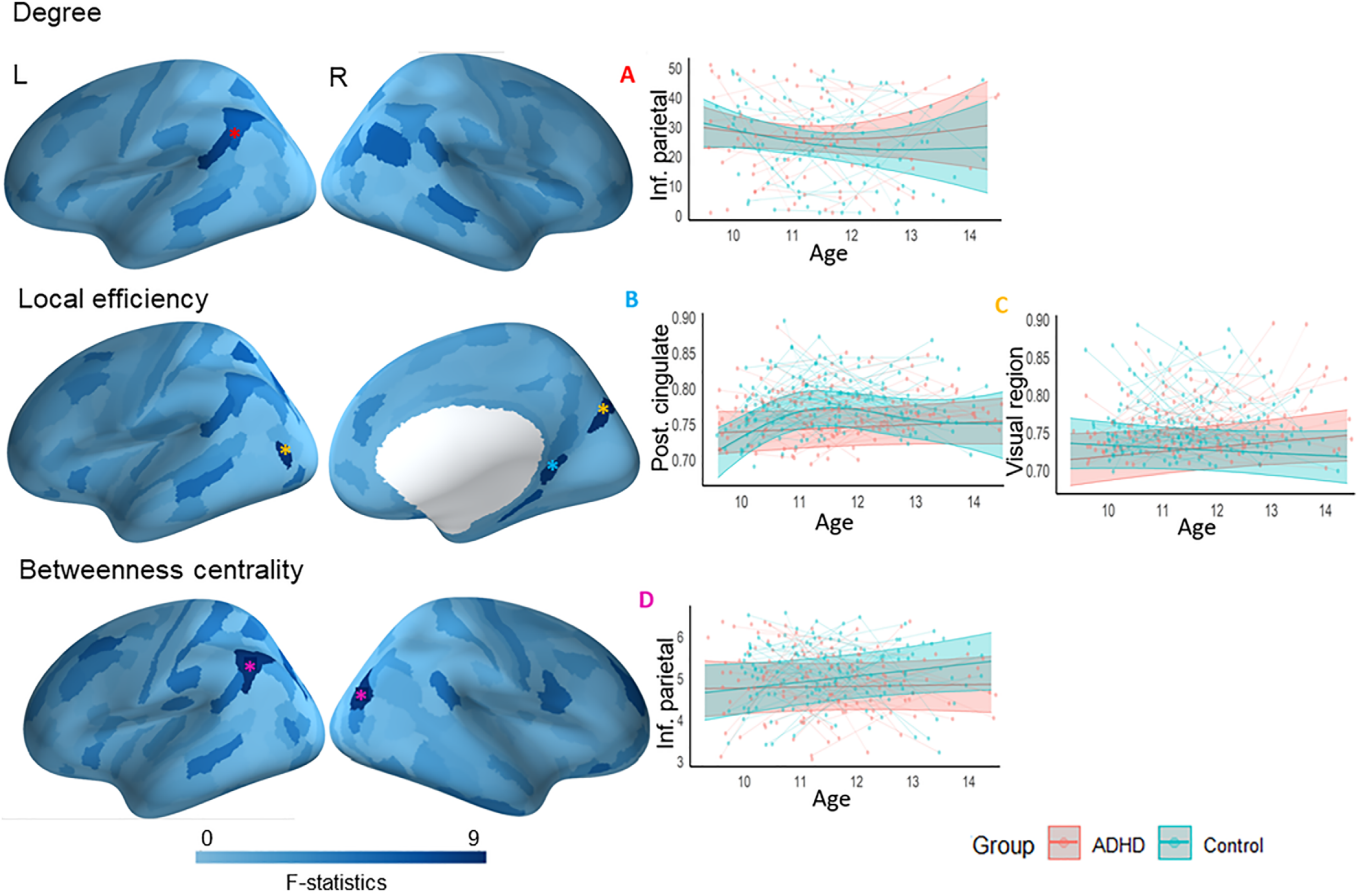
Group differences in developmental trajectories of structural connectivity (i.e., group x age interaction). “*” Indicates regions that survived FDR correction (*p* < 0.05). Inf. parietal, inferior parietal; post. cingulate, posterior cingulate. Plot A depicts the developmental trajectory of the brain regions marked with red * (left inf. parietal), and plot B depicts the developmental trajectory of the brain regions marked with blue * (right post. cingulate). Regions marked with yellow asterisk showed similar pattern of developmental trajectory (bilateral visual cortex) and plot C depicts the developmental trajectory of a brain region (left visual cortex) marked with yellow *. Regions marked with magenta asterisk showed similar pattern of developmental trajectory (left inf. parietal and dorsal visual cortex) and plot D depicts the developmental trajectory of a brain region (left inf. parietal) marked with magenta.

Children with ADHD showed a greater increasing trajectory of degree, relative to typically developing children, in the left inferior parietal cortex from late childhood to early adolescence (Figure 4 and Table S3). For LE, typically developing children showed decreased trajectory in the bilateral dorsal visual cortex whereas children with ADHD showed a stable pattern of structural connectivity. Further, typically developing children showed a non‐linear pattern of LE in the right dorsal posterior cingulate cortex (BA 31) characterized by an increase from 9 to 11 years and subsequent stabilisation to 14 years, whereas children with ADHD did not change across 9–14 years (Figure 4 and Table S3). Finally, typically developing children showed increased BC in the left inferior parietal cortex, and dorsal visual cortex whereas children with ADHD did not change between 9 and 14 years (Figure 4 and Table S3).

## 4 DISCUSSION

The goal of the current study was to investigate longitudinal changes in the brain's functional and structural topology in children with ADHD. Using graph theory analysis, our results indicate differences in the development of network degree, local efficiency and betweenness centrality predominantly in higher‐order cognitive and sensory regions such as anterior cingulate, posterior cingulate, frontal opercular cortex, superior temporal gyrus, inferior parietal cortex and visual regions in children with ADHD compared to typically developing children.

### 4.1 Group differences in functional cortical connectivity

The current findings highlight differential regional organization of functional networks in children with ADHD compared to their typically developing counterparts. Our analysis of functional connectivity showed group differences in anterior cingulate, inferior parietal cortex, and temporal cortex for degree, local efficiency and betweenness centrality. In addition, visual region was found to have an important role in degree where children with ADHD showed lower degree in visual region relative to typically developing children. Regions with consistent differences in degree, local efficiency and betweenness centrality mostly overlapped with the components of DMN, SAL and DAN. Similar brain regions were observed for different graph measures, and this could be either due to the location of these regions or the network measures might be correlated with each other. This suggest that these regions act as network hubs, with a large numbers of connections to other regions in the brain, and falling on the shortest path length between connected pairs of regions in the cortex (Fornito et al., 2016). Degree and BC show how strongly information flows across other network hubs, providing an indication of functional integration whereas LE gives information about how strongly regions are interconnected or segregated with each other. Disrupted degree and LE in the DMN, SAL, and DAN in ADHD have been previously reported in a range of cross‐sectional studies (Chen et al., 2019; Di Martino et al., 2013; Wang et al., 2020; Xia et al., 2014). In this study, lower degree was observed in children with ADHD (relative to typically developing children) in the bilateral inferior parietal, superior temporal and left visual cortices, which overlap with the DMN, SAL and visual networks and suggest that functional integration or information flow across these network hubs are affected in children with ADHD. Lower connectivity in these regions in children with ADHD has been previously reported and suggested as a reason for dysfunctions of attention and impulsivity observed in ADHD (Ana et al., 2010; Cortese et al., 2021; Wang et al., 2020; Zhang et al., 2020). We also observed higher degree in the left anterior cingulate, a key region of SAL that is responsible for higher‐level functions such as decision making, attention and emotion regulation. Previous studies have shown that higher connectivity in anterior cingulate is correlated with symptoms of inattention in children with ADHD (Cao, Shu, et al., 2014). We observed lower LE in ADHD (decreased segregation) in the right inferior parietal and insular cortex. These are key nodes of DMN and SAL responsible for various higher‐order cognitive functions, which aligns with prior literature showing aberrant connectivity in the attention system in children with ADHD (Tamm et al., 2006; Wang et al., 2009). Higher LE observed in children with ADHD in the left inferior temporal, right precuneus and ventral anterior cingulate cortices has been previously reported in ADHD (Wang et al., 2009).

Regarding betweenness centrality, prior functional connectivity studies have observed no significant difference in betweenness centrality (Kyeong et al., 2017; Tao et al., 2017; Wang et al., 2019). However, one functional connectivity study identified BC in the temporal, parietal and frontal regions of the brain as a strong predictor in classifying children with ADHD and typically developing controls (dos Santos et al., 2014). Lower BC observed in left inferior temporal cortex, a region overlapping with DAN, indicates decreased integration across this hub in children with ADHD relative to typically developing children. DAN is commonly implicated in children with ADHD and disrupted functional connectivity in DAN has been associated with attention deficit in ADHD (Konrad et al., 2006). Higher BC in right inferior parietal cortex, a region overlapping with DMN, suggests that inferior parietal cortex is highly used in children with ADHD for functional communication. Prior findings suggest that the abnormal functional communication of the DMN, attention networks and SAL affect various goal‐directed behaviours and could be a primary reason for the dysfunctions observed in children with ADHD (Corbetta & Shulman, 2002; Cortese et al., 2021; Lee, 2021; McCormick & Telzer, 2018; Menon, 2011; Supekar & Menon, 2012; Vossel et al., 2014). A network with small‐world properties shows balance between integration and segregation. Importantly, our findings of more decreased integration and segregation in the regions overlapping with DMN, SAL, DAN and visual network shows that the balance of functional network organization across these regions are affected in children with ADHD.

### 4.2 Differential developmental trajectories of graph measures in functional cortical connectivity

We also observed differential development of degree and local efficiency for intrinsic functional cortical connectivity predominantly in the DMN, SMN and visual networks. Typically developing children showed either a decreasing trajectory or minimal change in graph metrics for higher‐order cognitive and visual regions, which may reflect a normative pattern of development in functional connectivity between these networks (Nagel et al., 2011; Silk et al., 2016). In higher‐order and visual regions where typically developing children showed decreases in graph metrics, however, children with ADHD showed comparatively flat developmental trajectories, indicating minimal change in graph metrics over this period. Further, in other cognitive regions such as the superior temporal gyrus, which is responsible for social cognition and language processing, children with ADHD showed increasing trajectories of graph metrics compared to their peers. Disrupted functional connectivity in higher order cognitive networks and visual network in children with ADHD relative to typically developing children have been recently reported in our work (Soman et al., 2022), suggesting this may represent aberrant or potentially delayed maturation in ADHD. The difference in trajectories observed in the temporal, parietal and occipital regions fits within the literature of ADHD (Chen et al., 2019; Di Martino et al., 2013; Jiang et al., 2019; Marcos‐Vidal et al., 2018; Menon, 2011; Qian et al., 2019; Soman et al., 2022; Wang et al., 2009; Xia et al., 2014) and this may reflect that the functional neural circuits associated with attention, executive functions, fine motor control and visual perception are disrupted across development in children with ADHD.

### 4.3 Group differences in structural connectivity

Regionally, posterior cingulate and middle temporal gyrus exhibited group differences in degree, local efficiency, and betweenness centrality. In addition, frontal operculum showed an important role for degree. Again, similar brain regions observed for different graph measures could be either due to the location of these regions or correlation between the network measures (Fornito et al., 2016). None of the studies that examined graph properties of structural connectivity in children with ADHD used degree centrality and betweenness centrality to compute the topological difference between the groups (Beare et al., 2017; Cao et al., 2013; Griffiths et al., 2021; Qian et al., 2021). We observed higher degree in the right dorsal posterior cingulate and right frontal opercular cortex in children with ADHD compared to typically developing children. One study that used graph measures to examine the difference in grey matter organization between the groups observed lower degree centrality in dorsal posterior cingulate in ADHD group compared to control group (Griffiths et al., 2016). This contradicting result could be due to the difference in the structural measure used to compute graph measures. Higher degree in right dorsal posterior cingulate and frontal opercular cortex suggest the importance of these regions involved in integrating information across other networks in children with ADHD relative to typically developing children. Beare and colleagues (Beare et al., 2017) observed stronger connectivity in the subnetwork distributed broadly across the brain in children with ADHD and this was associated with the symptom severity of the disorder. Lower local efficiency or decreased segregation in the frontal, parietal (Cao et al., 2013) and temporal (Justina et al., 2015) regions have been previously reported, with studies suggesting this could be the reason for attention and executive deficits (Zhan et al., 2017) in children with ADHD. Interestingly, diffusion tensor imaging studies have reported abnormalities in white matter tracts connecting corpus callosum and temporal regions (cingulum bundle and superior longitudinal fasciculus II) which is associated with attention and executive deficits in children with ADHD (Aoki et al., 2018; Makris et al., 2008; Wu et al., 2017). Lower BC observed in inferior parietal cortex indicates decreased integration across this network hub in children with ADHD (Sporns, 2013). Early studies have shown that disruption in structural connectivity of hub regions could lead to aberrant functional connectivity (Sporns, 2013), which may account for the disrupted BC for functional connectivity we observed in a similar region. Again, higher BC in the right middle temporal cortex in children with ADHD indicates that middle temporal cortex is highly used for information transfer in ADHD. Consistent abnormalities reported in the frontal, parietal and temporal regions of the brain in a range of structural studies suggests that these regions are strongly affected in the pathophysiology of ADHD (Makris et al., 2008; Overmeyer et al., 2001; Wu et al., 2017). Collectively, the findings indicate that the structural abnormalities observed in the brain regions involved in integration and segregation across and between various frontal, temporal and parietal regions may be one explanation for the various higher order cognitive dysfunctions in children with ADHD. However, future studies examining the association between structural abnormalities and neurocognitive measures in children with ADHD are required to explore this further.

### 4.4 Differential developmental trajectories of graph measures in structural connectivity

We also found differential development of degree, local efficiency and betweenness centrality for structural connectivity predominantly in the parietal and occipital regions of the brain. Specifically, in higher‐order cognitive and visual regions where typically developing children showed either a minimal change or decrease, children with ADHD comparatively showed an increase in graph metrics across late childhood and early adolescence. Further, in other cognitive regions such as the posterior cingulate and inferior parietal cortex typically developing children showed a non‐linear and an increasing pattern of development whereas children with ADHD showed no change of graph metrics relative to their peers over this period. Prior research has identified structural abnormalities in the parietal and occipital regions in children with ADHD (Beare et al., 2017; Cao et al., 2013; Justina et al., 2015). Structural studies show that various regions of the brain undergo different developmental trajectories in typically developing children (Moore & Xia, 2022; Shaw et al., 2008), where sensory and visual regions undergo an early maturation and higher order cognitive regions undergo a protracted course of development. The increasing, decreasing or non‐linear pattern of developmental trajectory observed in typically developing children indicates the normative development of these regions across late childhood and early adolescence (Barnea‐Goraly et al., 2005; Sowell et al., 1999). Abnormal structural connections in the later maturing white matter tracts such as fronto‐parietal pathways have been previously demonstrated (Nagel et al., 2011), with studies suggesting this could be due to delayed or reduced myelination in ADHD. Collectively, the differential development of structural connectivity in ADHD group may indicate that the regions involved in transmitting neural signals across and between parietal and visual regions are disrupted in ADHD during childhood and adolescence.

Prior research using graph measures have typically used cross‐sectional samples and focused on either structural or functional connectivity (Cao et al., 2013; Cao, Wang, et al., 2014; Griffiths et al., 2021; Justina et al., 2015). There has been only one cross‐sectional study that examined differences in graph measures in children with ADHD relative to typically developing controls using both structural and functional connectivity (Bos et al., 2017). However, they did not observe any significant group differences in structural connectivity. The authors speculate that this could be due to the small sample size (*N* = 69) used in their study and has highlighted the importance of adequately powered longitudinal data to understand the changes in structural and functional development in children with ADHD (Bos et al., 2017). Our goal was to extend this research by examining maturation of the topology of both structural and functional connectome in a longitudinal cohort. Both functional and structural connectivity showed significant group differences and differential developmental trajectories for degree, local efficiency, and betweenness centrality predominantly in the higher‐order cognitive and visual regions. The regions observed for both the modalities for each graph measure are not identical but they seem to show similar networks of the brain, suggesting these might be strongly affected in the pathophysiology of the disorder. Structural and functional abnormalities in higher‐order cognitive and sensory regions of the brain could be the reason for various dysfunctions in sensory, cognitive and behavioural control observed in children with ADHD (Bos et al., 2017; Cao et al., 2013; Griffiths et al., 2021). However, future longitudinal research is required to determine the degree to which the developmental abnormalities in structure and function we have observed in ADHD might contribute to dysfunctions in sensory, cognitive and behavioural control.

Our study should be examined in light of some limitations. First, an important consideration is that structural and functional abnormalities are likely to be related to one another, and we did not investigate this relationship between two modalities. This represents an important future step to further examine the relationship between the topological measures of functional and structural connectomes, and how joint changes in structural and functional connectivity may be associated with changes in neurocognitive functioning or symptoms. Second, the study used only the basic graph measures that are predominantly identified from cross‐sectional studies in children with ADHD, and further research using other graph measures are needed to improve our knowledge about the development of functional and structural topology in ADHD. Third, the study has examined functional and structural alterations only in cortical brain regions, and hence future studies examining functional and structural connectivity alterations in subcortical brain regions could add knowledge regarding the development of subcortical regions in ADHD. Fourth, the observed functional and structural changes were not examined in relation to particular neurocognitive measures. There could be a lot of heterogeneity both in terms of behaviour and underlying brain structure/function among patients with ADHD. Further research is therefore needed to examine how these changes in functional and structural connectivity may be associated with changes in neurocognitive functioning and changes or remission of symptoms. Fifth, the study has investigated longitudinal changes in children between the ages of 9–14 years, and further studies are needed to understand the changes that could also occur earlier or later than that developmental period. Finally, whilst medication status was accounted for in the modelling, we did not specifically examine differences in trajectories between medication and non‐medication individuals with ADHD, due to the small number of individuals taking medication. Future studies investigating the differences in trajectories of structural and functional connectome between medicated and non‐medicated individuals with ADHD is required to explore the changes that might be caused in structural and functional connections due to medication. In summary, our study demonstrated topology of functional and structural connectomes that mature differently between typically developing controls and children with ADHD across childhood and adolescence. In particular, similar networks of the brain, predominantly featuring higher‐order cognitive and sensory regions, were affected in the functional and structural topology of children with ADHD relative to typically developing children, providing converging evidence that structural and functional connectivity in these cortical regions are strongly implicated in children with ADHD. However, it remains to be still investigated how the association between structural and functional cortical connectivity is affected in children with ADHD across childhood to adolescence.

## FUNDING INFORMATION

The study was funded by the National Health and Medical Research Council of Australia (NHMRC; project grants #1008522 and #1065895), a grant from the Collier Foundation. SS was supported by Postgraduate Research Scholarship from Deakin University (DUPRS).

## CONFLICT OF INTEREST STATEMENT

The authors declare no conflict of interest.

## Supporting information


**DATA S1.** Supporting information.

## Data Availability

The authors confirm that the data supporting the findings of this study are available within the article/its supplementary materials.
