# Supplementary material for: Functional and structural brain network development in children with attention deficit hyperactivity disorder
Source: Hum Brain Mapp. 2023 Mar 29;44(8):3394–409. doi: 10.1002/hbm.26288 (PMC10171546; doi:10.1002/hbm.26288)
Supplement: Supplementary file 1 — DATA S1. Supporting information. [file HBM-44-3394-s001.docx]

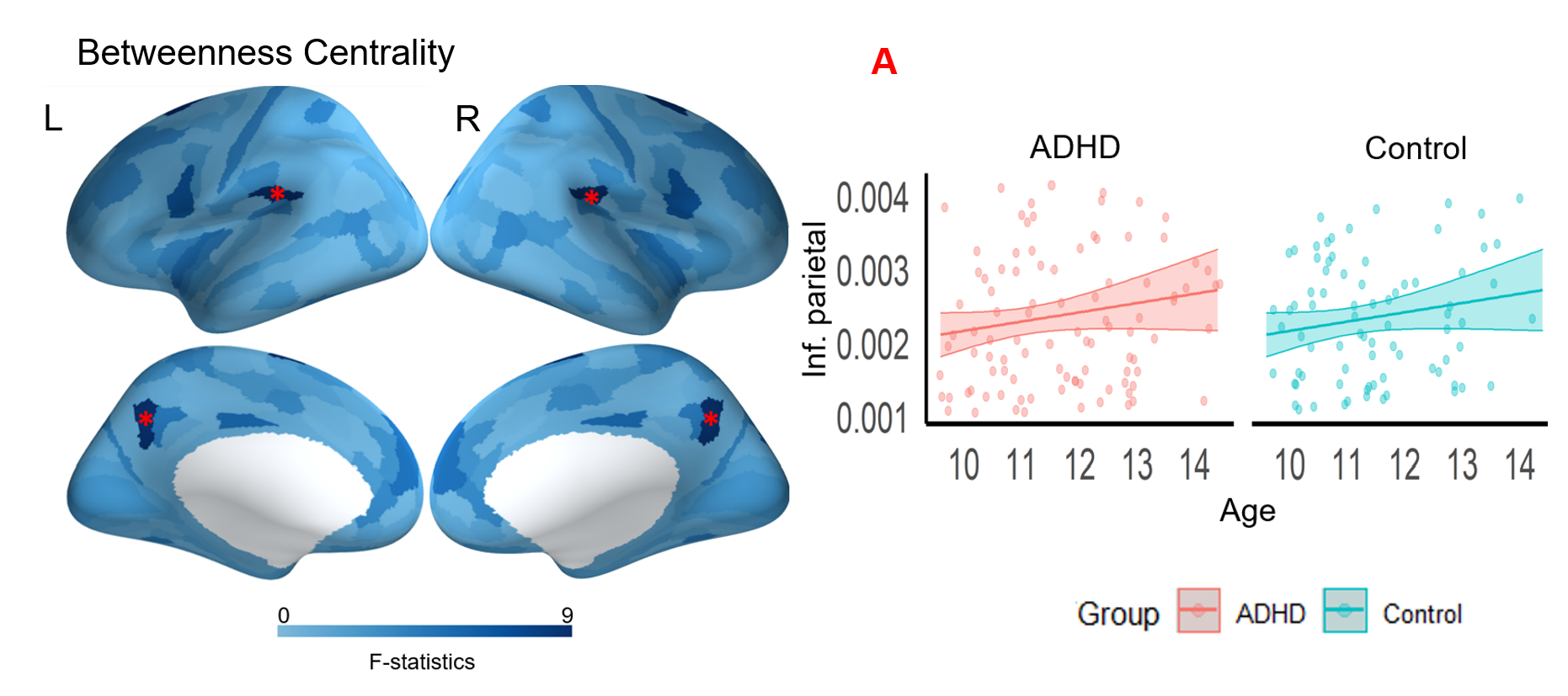


*Figure S1*. Developmental trajectories of functional connectivity (main effect of age). * Indicates regions that survived FDR correction(*p*<0.05). Inf. parietal – inferior parietal. Regions marked with red asterisk showed similar pattern of developmental trajectory (bilateral inf. parietal and precuneus) and plot A depicts the developmental trajectory of a brain region (left inf. parietal) marked with red * .


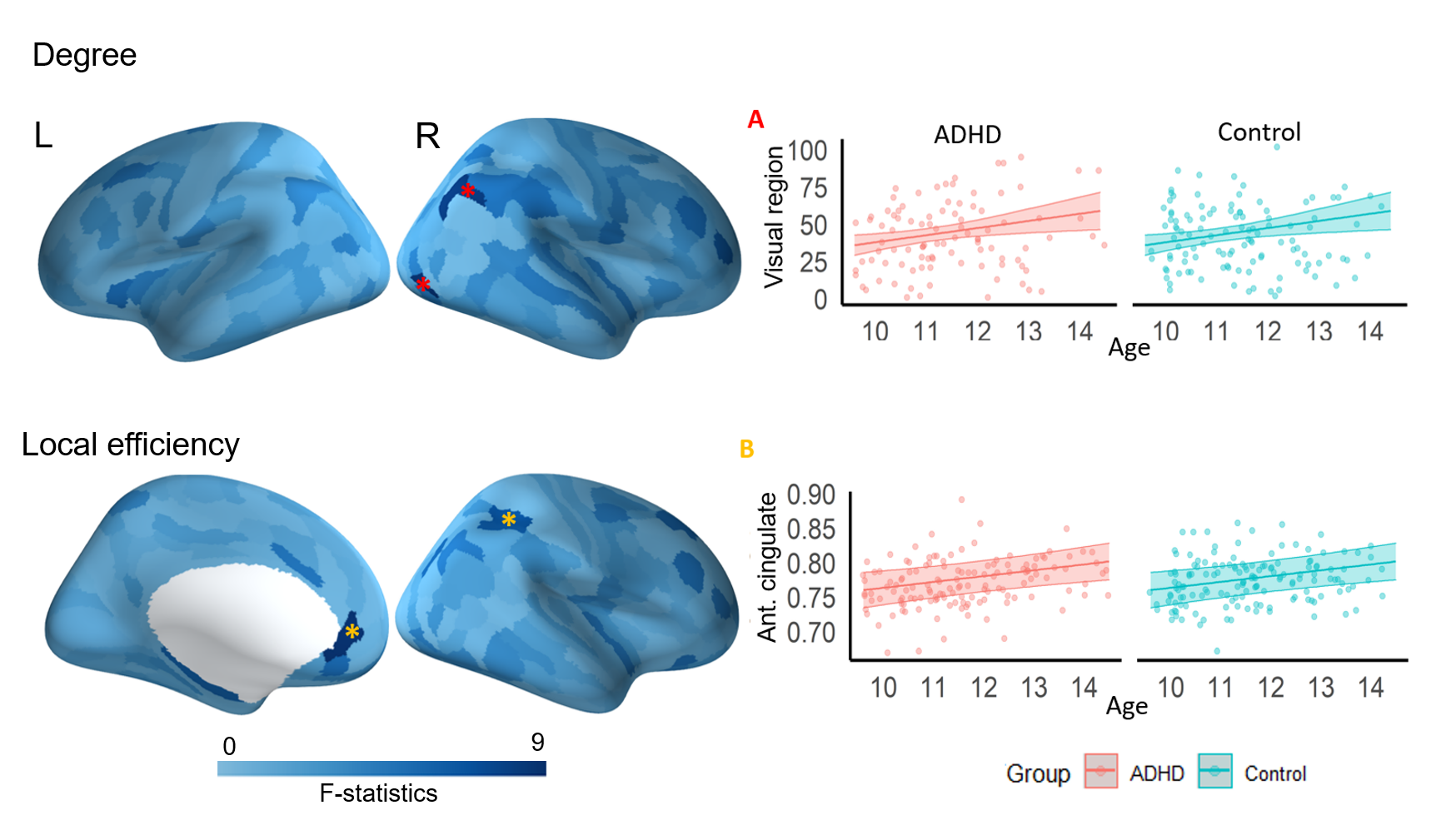


*Figure S2*. Developmental trajectories of structural connectivity (main effect of age). * Indicates regions that survived FDR correction(*p*<0.05). Ant. cingulate – anterior cingulate. Regions marked with red asterisk showed similar pattern of developmental trajectory (right inferior parietal and visual region) and plot A depicts the developmental trajectory of a brain region (right visual region) marked with red *. Regions marked with yellow asterisk showed similar pattern of developmental trajectory (left ant. cingulate and right superior parietal cortex) and plot B depicts the developmental trajectory of a brain region marked with yellow * (left ant.cingulate).


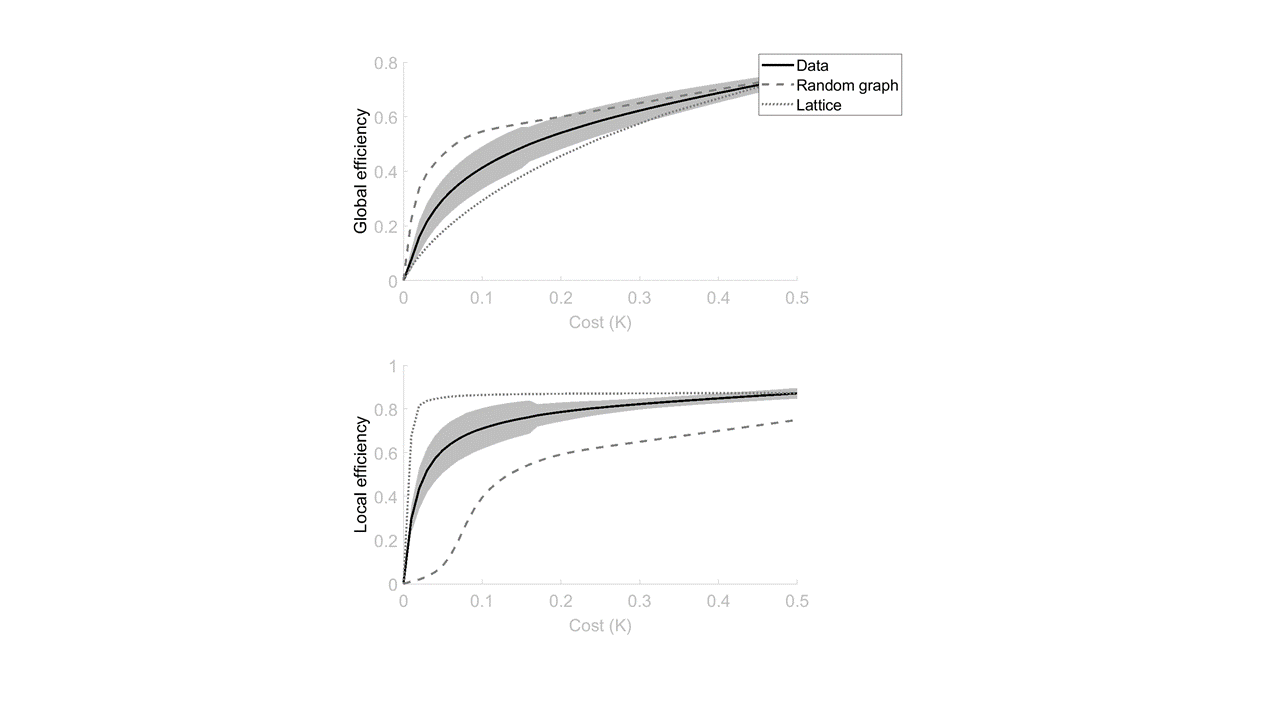


*Figure S3.* Plot showing different cost threshold values for global and local efficiency compared to lattice and random graph. This figure suggested that 0.15 was okay and higher thresholds result in too fragmented network (1, 2). Based on prior research that have used 0.15 we chose this as our threshold.

*Table S1.* Statistics for developmental trajectories of functional and structural connectivity (main effect of age)

| Connectivity | Graph measure | Regions | edf | Ref.df | F | p-value | FDR |
| --- | --- | --- | --- | --- | --- | --- | --- |
|  | Betweenness centrality | L inferior parietal cortex | 1.00 | 1.00 | 7.57 | 0.005 | 0.036 |
| Functional |  | R inferior parietal cortex | 1.00 | 1.00 | 7.86 | 0.005 | 0.033 |
|  |  | L precuneus | 1.00 | 1.00 | 8.34 | 0.004 | 0.024 |
|  |  | R precuneus | 1.00 | 1.00 | 8.24 | 0.004 | 0.026 |
|  | Degree | R angular gyrus | 1.00 | 1.00 | 7.52 | 0.006 | 0.035 |
|  |  | R visual cortex | 1.00 | 1.00 | 7.05 | 0.007 | 0.041 |
| Structural | Local efficiency | L anterior cingulate | 1.00 | 1.00 | 7.75 | 0.005 | 0.032 |
|  |  | R superior parietal cortex | 1.00 | 1.00 | 7.12 | 0.007 | 0.040 |
|  | Betweenness centrality | R angular gyrus | 1.00 | 1.00 | 8.17 | 0.004 | 0.025 |

NB: L - left hemisphere and R - right hemisphere

*Table S2.* Group differences between ADHD and control in functional and structural connectivity

| Connectivity | Graph measure | Regions | Estimate | Std.error | t-value | p-value | FDR |
| --- | --- | --- | --- | --- | --- | --- | --- |
|  | Degree | L inferior parietal cortex | 10.30 | 3.80 | 2.74 | 0.005 | 0.024 |
|  |  | R inferior parietal cortex | 10.35 | 3.75 | 2.77 | 0.005 | 0.022 |
|  |  | L superior temporal gyrus | 10.60 | 3.73 | 2.84 | 0.004 | 0.020 |
|  |  | R superior temporal gyrus | 9.88 | 4.02 | 2.64 | 0.007 | 0.041 |
|  |  | L visual cortex | 10.31 | 3.79 | 2.71 | 0.006 | 0.032 |
| Functional |  | L anterior cingulate | -11.13 | 3.52 | -3.00 | 0.001 | 0.010 |
|  | LE | R inferior parietal cortex | 0.02 | 0.008 | 2.85 | 0.004 | 0.020 |
|  |  | R insula | 0.01 | 0.007 | 2.74 | 0.006 | 0.030 |
|  |  | L inferior temporal cortex | -0.02 | 0.009 | -2.84 | 0.004 | 0.022 |
|  |  | R precuneus | -0.02 | 0.008 | -2.80 | 0.004 | 0.025 |
|  |  | R anterior cingulate | 0.02 | 0.01 | -2.62 | 0.008 | 0.044 |
|  | BC | L inferior temporal cortex | -0.01 | 0.005 | 2.96 | 0.003 | 0.013 |
|  |  | R inferior parietal cortex | -0.02 | 0.005 | -2.71 | 0.006 | 0.032 |
|  | Degree | R dorsal posterior cingulate | -7.31 | 3.18 | -2.75 | 0.006 | 0.031 |
|  |  | R frontal opercular cortex | -7.19 | 3.12 | -2.73 | 0.006 | 0.033 |
| Structural | LE | R dorsal posterior cingulate | 0.01 | 0.005 | 2.86 | 0.004 | 0.020 |
|  |  | R middle temporal cortex | 0.01 | 0.005 | 2.85 | 0.004 | 0.020 |
|  | BC | R angular gyrus | 0.64 | 0.22 | 2.87 | 0.004 | 0.020 |
|  |  | R middle temporal cortex | -0.32 | 0.12 | -2.74 | 0.006 | 0.030 |

NB: L - left hemisphere and R - right hemisphere

*Table S3.* Statistics for differential developmental trajectories of functional and structural connectivity

(group x age interaction)

| Connectivity | Graph measure | Regions | edf | Ref.df | F | p-value | FDR |
| --- | --- | --- | --- | --- | --- | --- | --- |
|  | Degree | R superior temporal gyrus | 1.00 | 1.00 | 9.40 | 0.003 | 0.044 |
|  |  | Left precuneus | 1.00 | 1.00 | 11.32 | 0.001 | 0.020 |
| Functional | Local efficiency | L visual cortex | 1.00 | 1.00 | 10.24 | 0.002 | 0.035 |
|  |  | R visual cortex | 1.00 | 1.00 | 10.26 | 0.002 | 0.034 |
|  |  | R subiculum | 1.00 | 1.00 | 11.13 | 0.001 | 0.022 |
|  | Degree | L inferior parietal cortex | 1.00 | 1.00 | 8.45 | 0.004 | 0.025 |
| Structural | Local efficiency | L visual cortex | 1.00 | 1.00 | 8.35 | 0.004 | 0.027 |
|  |  | R visual cortex | 1.00 | 1.00 | 8.21 | 0.004 | 0.029 |
|  |  | R dorsal posterior cingulate | 2.76 | 2.95 | 7.05 | 0.006 | 0.045 |
|  | Betweenness centrality | L inferior parietal cortex | 1.00 | 1.00 | 8.31 | 0.004 | 0.028 |
|  |  | L visual cortex | 1.00 | 1.00 | 8.15 | 0.004 | 0.029 |

NB: L - left hemisphere and R - right hemisphere

*Table S4.* Group differences between ADHD and control in functional and structural connectivity, threshold – 0.1

| Connectivity | Graph measure | Regions | Estimate | Std.error | t-value | p-value | FDR |
| --- | --- | --- | --- | --- | --- | --- | --- |
|  | Degree | L inferior parietal cortex | 8.15 | 5.85 | 2.43 | 0.015 | 0.052 |
|  |  | R inferior parietal cortex | 10.28 | 3.73 | 2.76 | 0.006 | 0.033 |
|  |  | L superior temporal gyrus | 9.45 | 4.34 | 2.42 | 0.009 | 0.050 |
|  |  | R superior temporal gyrus | 8.76 | 5.63 | 2.33 | 0.026 | 0.055 |
|  |  | L visual cortex | 10.38 | 3.68 | 2.82 | 0.005 | 0.028 |
| Functional |  | L anterior cingulate | -11.03 | 3.61 | -2.95 | 0.004 | 0.032 |
|  | LE | R inferior parietal cortex | 0.03 | 0.007 | 2.88 | 0.003 | 0.020 |
|  |  | R insula | 0.01 | 0.009 | 2.35 | 0.009 | 0.050 |
|  |  | L inferior temporal cortex | -0.02 | 0.009 | -2.82 | 0.004 | 0.025 |
|  |  | R precuneus | -0.01 | 0.006 | -2.88 | 0.003 | 0.022 |
|  |  | R anterior cingulate | 0.01 | 0.01 | -2.65 | 0.008 | 0.047 |
|  | BC | L inferior temporal cortex | -0.01 | 0.008 | 2.45 | 0.006 | 0.045 |
|  |  | R inferior parietal cortex | -0.01 | 0.007 | -2.67 | 0.008 | 0.048 |
|  | Degree | R dorsal posterior cingulate | -7.20 | 3.34 | -2.54 | 0.009 | 0.047 |
|  |  | R frontal opercular cortex | -7.05 | 3.44 | -2.53 | 0.009 | 0.048 |
| Structural | LE | R dorsal posterior cingulate | 0.01 | 0.007 | 2.75 | 0.005 | 0.035 |
|  |  | R middle temporal cortex | 0.01 | 0.008 | 2.73 | 0.006 | 0.038 |
|  | BC | R angular gyrus | 0.54 | 0.42 | 2.77 | 0.006 | 0.040 |
|  |  | R middle temporal cortex | -0.25 | 0.52 | -2.54 | 0.009 | 0.045 |

NB: L - left hemisphere and R - right hemisphere

*Table S5.* Statistics for differential developmental trajectories of functional and structural connectivity

(group x age interaction), threshold – 0.1

| Connectivity | Graph measure | Regions | edf | Ref.df | F | p-value | FDR |
| --- | --- | --- | --- | --- | --- | --- | --- |
|  | Degree | R superior temporal gyrus | 1.00 | 1.00 | 9.52 | 0.002 | 0.040 |
|  |  | Left precuneus | 1.00 | 1.00 | 9.75 | 0.005 | 0.045 |
| Functional | Local efficiency | L visual cortex | 1.00 | 1.00 | 10.02 | 0.004 | 0.043 |
|  |  | R visual cortex | 1.00 | 1.00 | 9.26 | 0.006 | 0.048 |
|  |  | R subiculum | 1.00 | 1.00 | 11.23 | 0.001 | 0.020 |
|  | Degree | L inferior parietal cortex | 1.00 | 1.00 | 8.20 | 0.006 | 0.040 |
| Structural | Local efficiency | L visual cortex | 1.00 | 1.00 | 7.85 | 0.006 | 0.047 |
|  |  | R visual cortex | 1.00 | 1.00 | 8.25 | 0.003 | 0.025 |
|  |  | R dorsal posterior cingulate | 2.76 | 2.95 | 7.00 | 0.008 | 0.050 |
|  | Betweenness centrality | L inferior parietal cortex | 1.00 | 1.00 | 8.20 | 0.005 | 0.030 |
|  |  | L visual cortex | 1.00 | 1.00 | 8.15 | 0.004 | 0.029 |

NB: L - left hemisphere and R - right hemisphere

*Table S6.* Group differences between ADHD and control in functional and structural connectivity, threshold – 0.2

| Connectivity | Graph measure | Regions | Estimate | Std.error | t-value | p-value | FDR |
| --- | --- | --- | --- | --- | --- | --- | --- |
|  | Degree | L inferior parietal cortex | 9.33 | 4.43 | 2.50 | 0.009 | 0.045 |
|  |  | R inferior parietal cortex | 9.50 | 4.23 | 2.755 | 0.008 | 0.044 |
|  |  | L superior temporal gyrus | 8.45 | 5.12 | 2.15 | 0.04 | 0.07 |
|  |  | R superior temporal gyrus | 8.50 | 5.33 | 2.20 | 0.03 | 0.06 |
|  |  | L visual cortex | 10.12 | 3.88 | 2.57 | 0.007 | 0.033 |
| Functional |  | L anterior cingulate | -10.20 | 3.76 | -2.87 | 0.003 | 0.035 |
|  | LE | R inferior parietal cortex | 0.01 | 0.01 | 2.13 | 0.04 | 0.08 |
|  |  | R insula | 0.01 | 0.009 | 2.35 | 0.009 | 0.050 |
|  |  | L inferior temporal cortex | -0.02 | 0.009 | -2.40 | 0.009 | 0.050 |
|  |  | R precuneus | -0.01 | 0.008 | -2.75 | 0.005 | 0.048 |
|  |  | R anterior cingulate | 0.01 | 0.01 | -2.32 | 0.035 | 0.053 |
|  | BC | L inferior temporal cortex | -0.01 | 0.01 | 2.11 | 0.043 | 0.055 |
|  |  | R inferior parietal cortex | -0.01 | 0.007 | -2.10 | 0.049 | 0.067 |
|  | Degree | R dorsal posterior cingulate | -7.05 | 3.28 | -2.45 | 0.009 | 0.050 |
|  |  | R frontal opercular cortex | -6.75 | 3.56 | -2.42 | 0.015 | 0.060 |
| Structural | LE | R dorsal posterior cingulate | 0.01 | 0.008 | 2.78 | 0.007 | 0.045 |
|  |  | R middle temporal cortex | 0.01 | 0.008 | 2.73 | 0.006 | 0.038 |
|  | BC | R angular gyrus | 0.25 | 0.62 | 2.22 | 0.035 | 0.054 |
|  |  | R middle temporal cortex | -0.20 | 0.57 | -2.44 | 0.010 | 0.052 |

*Table S7.* Group differences between ADHD and control in functional and structural connectivity, threshold – 0.2

| Connectivity | Graph measure | Regions | edf | Ref.df | F | p-value | FDR |
| --- | --- | --- | --- | --- | --- | --- | --- |
|  | Degree | R superior temporal gyrus | 1.00 | 1.00 | 8.25 | 0.035 | 0.054 |
|  |  | Left precuneus | 1.00 | 1.00 | 9.65 | 0.006 | 0.048 |
| Functional | Local efficiency | L visual cortex | 1.00 | 1.00 | 8.12 | 0.034 | 0.065 |
|  |  | R visual cortex | 1.00 | 1.00 | 9.06 | 0.008 | 0.050 |
|  |  | R subiculum | 1.00 | 1.00 | 10.76 | 0.003 | 0.035 |
|  | Degree | L inferior parietal cortex | 1.00 | 1.00 | 7.89 | 0.044 | 0.063 |
| Structural | Local efficiency | L visual cortex | 1.00 | 1.00 | 7.56 | 0.008 | 0.050 |
|  |  | R visual cortex | 1.00 | 1.00 | 7.12 | 0.012 | 0.054 |
|  |  | R dorsal posterior cingulate | 2.66 | 2.85 | 6.72 | 0.015 | 0.055 |
|  | Betweenness centrality | L inferior parietal cortex | 1.00 | 1.00 | 7.85 | 0.007 | 0.045 |
|  |  | L visual cortex | 1.00 | 1.00 | 8.05 | 0.006 | 0.044 |

NB: L - left hemisphere and R - right hemisphere

*Table S8.* Group differences between ADHD and control in functional and structural connectivity (excluding medicated subjects)

| Connectivity | Graph measure | Regions | Estimate | Std.error | t-value | p-value | FDR |
| --- | --- | --- | --- | --- | --- | --- | --- |
|  | Degree | L inferior parietal cortex | 9.87 | 3.50 | 2.54 | 0.008 | 0.044 |
|  |  | R inferior parietal cortex | 10.12 | 3.65 | 2.68 | 0.006 | 0.032 |
|  |  | L superior temporal gyrus | 10.65 | 3.75 | 2.98 | 0.038 | 0.025 |
|  |  | R superior temporal gyrus | 9.57 | 4.00 | 2.62 | 0.008 | 0.044 |
|  |  | L visual cortex | 9.24 | 3.48 | 2.53 | 0.008 | 0.050 |
| Functional |  | L anterior cingulate | -11.13 | 3.52 | -3.00 | 0.001 | 0.010 |
|  | LE | R inferior parietal cortex | 0.02 | 0.008 | 2.85 | 0.004 | 0.020 |
|  |  | R insula | 0.01 | 0.007 | 2.74 | 0.006 | 0.030 |
|  |  | L inferior temporal cortex | -0.02 | 0.009 | -2.84 | 0.004 | 0.022 |
|  |  | R precuneus | -0.02 | 0.008 | -2.79 | 0.005 | 0.029 |
|  |  | R anterior cingulate | 0.01 | 0.03 | -2.60 | 0.009 | 0.050 |
|  | BC | L inferior temporal cortex | -0.01 | 0.005 | 2.94 | 0.003 | 0.015 |
|  |  | R inferior parietal cortex | -0.02 | 0.005 | -2.70 | 0.006 | 0.030 |
|  | Degree | R dorsal posterior cingulate | -7.30 | 3.15 | -2.73 | 0.006 | 0.030 |
|  |  | R frontal opercular cortex | -7.00 | 3.00 | -2.70 | 0.006 | 0.030 |
| Structural | LE | R dorsal posterior cingulate | 0.01 | 0.005 | 2.86 | 0.004 | 0.020 |
|  |  | R middle temporal cortex | 0.01 | 0.005 | 2.85 | 0.004 | 0.020 |
|  | BC | R angular gyrus | 0.65 | 0.20 | 3.00 | 0.004 | 0.020 |
|  |  | R middle temporal cortex | -0.22 | 0.32 | -2.65 | 0.007 | 0.043 |

*Table S9.* Statistics for differential developmental trajectories of functional and structural connectivity

(group x age interaction) (excluding medicated subjects)

| Connectivity | Graph measure | Regions | edf | Ref.df | F | p-value | FDR |
| --- | --- | --- | --- | --- | --- | --- | --- |
|  | Degree | R superior temporal gyrus | 1.00 | 1.00 | 9.43 | 0.003 | 0.042 |
|  |  | Left precuneus | 1.00 | 1.00 | 10.52 | 0.004 | 0.031 |
| Functional | Local efficiency | L visual cortex | 1.00 | 1.00 | 10.33 | 0.002 | 0.033 |
|  |  | R visual cortex | 1.00 | 1.00 | 9.66 | 0.005 | 0.050 |
|  |  | R subiculum | 1.00 | 1.00 | 11.32 | 0.001 | 0.020 |
|  | Degree | L inferior parietal cortex | 1.00 | 1.00 | 8.68 | 0.037 | 0.022 |
| Structural | Local efficiency | L visual cortex | 1.00 | 1.00 | 8.00 | 0.005 | 0.045 |
|  |  | R visual cortex | 1.00 | 1.00 | 8.45 | 0.004 | 0.023 |
|  |  | R dorsal posterior cingulate | 2.76 | 2.95 | 7.47 | 0.006 | 0.040 |
|  | Betweenness centrality | L inferior parietal cortex | 1.00 | 1.00 | 8.75 | 0.004 | 0.025 |
|  |  | L visual cortex | 1.00 | 1.00 | 8.28 | 0.004 | 0.022 |

**References**

1. Power JD, Fair DA, Schlaggar BL, Petersen SEJN (2010): The development of human functional brain networks. 67:735-748.

2. dos Santos Siqueira A, Biazoli Junior CE, Comfort WE, Rohde LA, Sato JR (2014): Abnormal functional resting-state networks in ADHD: graph theory and pattern recognition analysis of fMRI data. *BioMed research international*. 2014:380531.
